# CCR2 and CCR5 Expression on Tph Cells Is Associated with IL-21 and Renal Involvement in Systemic Lupus Erythematosus

**DOI:** 10.3390/diagnostics16213687

**Published:** 2026-11-08

**Authors:** Katya Rocío Félix-Murray, Jhonatan Velador-Mendoza, Miguel Marín-Rosales, Pablo C. Ortiz-Lazareno, Laura Perfinka Herrera-González, Diana Celeste Salazar-Camarena, Noemí Espinoza-García, Esther Guadalupe Corona-Sánchez, María Paulina Reyes-Mata, Héctor Giancarlo Torres-Nuño, Claudia Azucena Palafox-Sánchez

**Affiliations:** 1 Doctorado en Ciencias en Biología Molecular en Medicina, Centro Universitario de Ciencias de la Salud, Universidad de Guadalajara, Guadalajara 44340, Jalisco, Mexico; katya.felix@academicos.udg.mx; 2 Departamento de Disciplinas Filosófico, Metodológicas e Instrumentales, Centro Universitario de Ciencias de la Salud, Universidad de Guadalajara, Guadalajara 44340, Jalisco, Mexico; celeste.salazar@academicos.udg.mx (D.C.S.-C.); noemi.espinoza@academicos.udg.mx (N.E.-G.); esther.corona@academicos.udg.mx (E.G.C.-S.); paulina.reyes@academicos.udg.mx (M.P.R.-M.); 3 Doctorado en Ciencias Biomédicas, Centro Universitario de Ciencias de la Salud, Universidad de Guadalajara, Guadalajara 44340, Jalisco, Mexico; jhonatan.velador9888@alumnos.udg.mx; 4 Secretaría de Salud Jalisco, Hospital General de Occidente, Zapopan 45170, Jalisco, Mexico; miguel.marin@academicos.udg.mx; 5 Instituto de Investigación en Medicina Traslacional, Centro Universitario de Ciencias de la Salud, Universidad de Guadalajara, Guadalajara 44340, Jalisco, Mexico; giancarlo.torres@academicos.udg.mx; 6 Centro de Investigación Biomédica de Occidente (CIBO), Instituto Mexicano del Seguro Social, Guadalajara 44340, Jalisco, Mexico; pablolazareno@gmail.com; 7 Laboratorio de Patología Diagnóstica (LAPADI), Centro Universitario de Ciencias de la Salud, Universidad de Guadalajara, Guadalajara 44340, Jalisco, Mexico; perfinka.herrera@academicos.udg.mx; 8 Instituto de Biología Molecular en Medicina y Terapia Génica, Centro Universitario de Ciencias de la Salud, Universidad de Guadalajara, Guadalajara 44340, Jalisco, Mexico

**Keywords:** T peripheral helper, chemokine receptor 2, chemokine receptor 5, lupus nephritis, interleukin-21

## Abstract

**Background/Objectives**: The increased frequency of circulating T follicular helper (cTfh) and T peripheral helper (Tph) cells in patients with systemic lupus erythematosus (SLE) and their association with higher disease activity have been reported. However, their role in the immunopathogenesis of SLE remains unclear. CCR2 and CCR5 have been identified on the surface of cTfh and Tph cells in autoimmune diseases such as Sjögren’s Disease. **Methods**: This study evaluates the association between CCR2^+^CCR5^+^cTfh and CCR2^+^CCR5^+^Tph cell frequencies and serum cytokine levels in patients with SLE and lupus nephritis (LN). **Results**: SLE patients showed higher frequencies of cTfh and Tph cells. Overall, CCR2 and CCR5 expression was higher in Tph cells than in cTfh cells. The frequency of CCR5^+^Tph and CCR5^+^cTfh cells was higher in patients with proliferative LN than in those with mixed-type LN. IL-21 correlated with the frequency of CCR2^+^CCR5^+^ Tph cells. In renal tissue, expression of CD4, CXCR5, CCR5, and CCR2 was more prominent in tubulo-interstitial areas with predominantly chronic lesions. **Conclusions**: Our results provide information on the expression of CCR2 and CCR5 in cTfh and Tph cells in SLE and offer insights into the association with inducing cytokines, highlighting the potential role of these cells in LN.

## 1. Introduction

Systemic Lupus Erythematosus (SLE) is a chronic and systemic autoimmune disease affecting approximately 3.4 million people worldwide. It is characterized by inflammation and immune-mediated damage to multiple organ systems, including the kidneys, leading to lupus nephritis (LN) in 20 to 60% of SLE patients [1,2]. The etiology of SLE is multifactorial, involving environmental, genetic, epigenetic, hormonal, and immune-related risk factors. The participation of autoreactive T and B cells and their collaboration in the overproduction of autoantibodies play essential roles in SLE pathogenesis, leading to the formation and deposition of immunocomplexes in inflamed tissues [3]. Inflammation in the kidneys and other tissues can lead to the formation of lymphoid organ-like structures named tertiary lymphoid structures (TLS), which consist of diverse immune cells, including B and T cells [4]. In this context, follicular helper T (Tfh) cells, a CD4+ T cell subset that specializes in promoting B cell proliferation and differentiation into plasma cells to produce antibodies, have been investigated in TLS in SLE and other autoimmune diseases [5].

Tfh cells are a T helper subpopulation characterized by the expression of PD1, CXCR5, and ICOS in secondary lymphoid organs. In contrast, in the peripheral blood, similar populations are identified as circulating T follicular helper (cTfh) [6] and T peripheral helper (Tph) cells. Previous studies found that the frequencies of cTfh and Tph cells were increased and positively correlated with disease activity in SLE and other autoimmune diseases [7,8]. Tph cells lack CXCR5 expression but share effector capacities with cTfh cells, and both are key players in SLE pathogenesis by inducing plasma cell differentiation primarily through IL-21 production [7]. However, how these cTfh and Tph cells can arrive or arise in TLS is not fully understood. The expression of migration markers, such as the chemokine receptors CCR2 and CCR5, in these two circulating PD1^+^CD4^+^ T cell subsets has been proposed previously but still requires further investigation in the context of SLE and LN [9,10].

Therefore, in this study, we aimed to investigate the expression of CCR2 and CCR5 in cTfh and Tph cells and to evaluate the relationship with disease activity and serum cytokine levels in LN and SLE patients. These findings could offer new insights into understanding the immunopathogenesis of not only SLE and LN, but also other diseases that involve the migration of cTfh and Tph to inflamed tissues.

## 2. Materials and Methods

### 2.1. Study Design and Subjects

An analytical, cross-sectional, observational study was conducted from January 2024 to February 2025. Recruitment of 40 SLE patients was conducted through consecutive sampling during outpatient consultations in the Rheumatology Department of the Hospital General de Occidente. Patients were diagnosed according to the EULAR/ACR 2019 [11] criteria, and those who met the inclusion criteria, agreed to participate in the study, and signed informed consent were included. For the HC group, 30 participants, similarly in age and sex to the SLE group, without autoimmune diseases, cancer, or recent infections, were recruited. SLEDAI-2K and Mex-SLEDAI were used to assess disease activity. Subjects with SLE were divided into two strata: mildly active or nonactive disease (a SLEDAI-2K score of 5 or below) and clearly active disease (a SLEDAI-2K score higher than 5) [12,13]. Also, comparisons were made according to LN involvement. The complete clinical criteria used to define active kidney involvement (*n* = 15), included proteinuria thresholds, active urinary sediment, and GFR alterations, as well as the histological ISN/RPS classification for the biopsied sub-cohort (*n* = 9).

### 2.2. Frequency of T Helper Subpopulations by Flow Cytometry

Peripheral blood mononuclear cells (PBMCs) were isolated from 20 mL of EDTA-anticoagulated blood by density gradient centrifugation with a 1:1 volume ratio of Histopaque-1077 medium (Sigma-Aldrich, Merck, Darmstadt, Germany) at room temperature using a centrifuge (Heraeus Megafuge 8, ThermoFisher Scientific, Suzhou, China). Subsequently, the isolated PBMCs were washed 3 times with 10 mL of 1X phosphate-buffered saline (PBS). An aliquot of the cell suspension was taken for counting with 0.4% trypan blue viability staining, then resuspended in the appropriate volume of 1X PBS to adjust the concentration to 1× 10^6^ cells/mL. The cells were maintained in suspension in the same buffer under refrigeration (4 °C) until use for subsequent assays. The CytoFLEX V4-B5-R3 flow cytometer (BD, Brea, CA, USA) was used to determine the cell frequency. The PBMCs were used to determine the relative proportions of cTfh and Tph in SLE and HC by surface staining with fluorochrome-conjugated anti-human antibodies, as detailed in Supplementary Table S1. At least 5 × 10^5^ events of the previously stained cells were recorded. Flow cytometry data analysis was performed using FlowJo v10 (Becton, Dickinson, and Company, Franklin Lakes, NJ, USA) and the strategy outlined in Supplementary Figure S1.

### 2.3. Serum Concentration of Cytokines by ELISA

Blood serum was separated and stored frozen (−20 °C) until use. ELISA quantified the serum concentration of cytokines from patients and subjects according to the manufacturer’s instructions for IL-21 (433804/BioLegend Inc., San Diego, CA, USA), TGFB-3 (EH613RB/Invitrogen, Carlsbad, CA, USA), IL-6 (BMS213-2/Invitrogen, Carlsbad, CA, USA), IL-17 (D1700/R&Dsystems, Inc., Minneapolis, MN, USA) and CXCL13 (DCX130/R&D Systems, Inc., Minneapolis, CA, USA). All samples were analyzed in duplicate. Absorbance was measured using a MultiskanGo microplate reader (Thermo Scientific, Waltham, MA, USA), and optical density was read at 450 nm, with the 570 nm reading subtracted from the first read.

### 2.4. Immunohistochemical Staining, Image Processing, and Statistical Analysis of Kidney Tissues

We included nine kidney biopsies from patients with LN. To ensure temporal alignment between systemic and tissue immunophenotypes, peripheral blood samples were obtained within a median interval of 2, 1 days (median, IQR) from the renal biopsy. All cases were reviewed and classified by an expert nephropathologist according to the 2018 ISR/RPS classification for NL [14]. The tissues were fixed in 10% buffered formaldehyde and embedded in paraffin. Tissues were sectioned at 3 μm, mounted on electrocharged slides, and deparaffinized at 60 °C for 30 min. To rehydrate the kidney tissues, they were immersed in xylene using graded ethanol dilutions, then in distilled water, and finally in cold water for 10 min. Briefly, after tissue rehydration, epitope retrieval was performed at 90 °C for 30 min in 10 mM sodium citrate (pH 6) for CCR5, PD1, CXCR5, CCR2, and CD4. All slides were subjected to endogenous peroxidase blockade with 0.09% hydrogen peroxide for 5 min and washed with TBST for 4 min. Later, slides were incubated for 30 min with the following primary antibodies: human CCR5 antibody (monoclonal mouse IgG2B clone #45523, R&D Systems (Minneapolis, CA, USA), cat no. MAB181, dilution 1:100), human PD-1 antibody (monoclonal mouse IgG2A clone #1026563, R&D Systems (Minneapolis, CA, USA), cat no. MAB10865, dilution 1:100), human CXCR5 antibody (monoclonal mouse IgG2B clone #51505, R&D Systems, (Minneapolis, CA, USA), cat no. MAB190, dilution 1:100), human CCR2 antibody (monoclonal mouse IgG2B, R&D Systems (Minneapolis, CA, USA), cat no. MAB150, dilution 1:100) and human CD4 (monoclonal rabbit IgG, Biocare (Pacheco, CA, USA), clone EP204, dilution 1:100). Following incubation with primary antibodies and buffer washing, the slides were incubated for 10 min with the universal post-primary antibody (Novolink Max polymer Detection System (Leica Biosystems, Newcastle upon Tyne, NE12 8EW, United Kingdom), LOT 6128989). Diaminobenzidine (DAB) was used as a chromogen, producing a red-brown color in positive reactions, followed by water washing; staining with Gill’s Hematoxylin; additional water washing; dehydration with OH-ISO water; drying; assembly; and resin coating.

After tissue staining, we photographed each slide, capturing three images per zone (glomerular, tubular, and interstitial). To handle potential spatial clustering and within-biopsy dependence, measurements from all captured fields per zone (glomerular, tubular, or interstitial) were aggregated into a single mean value per patient. We used the OMAX light microscope at 4X, 10X, and 40X magnification. We used Fiji/ImageJ v151j8/software and the DAB deconvolution plugin (National Institutes of Health, Bethesda, MD, USA, https://imagej.net/ij/, accessed on 04 august 2026) to analyze the images and obtain the immunoreaction score. We evaluated pixel intensity in the previously acquired photos according to Chatterjee et al. [15]. Color deconvolution was performed to isolate the chromogen (DAB) signal. We established a fixed intensity threshold (intensity range: 125–255 in 8-bit grayscale) to identify positively stained tissue and exclude background staining. The percentage-positive area was calculated as the area occupied by positive DAB pixels divided by the total tissue area within the region of interest (ROI), excluding lumens and blank background, which served as the denominator. This metric quantifies the spatial extent of positivity within the tissue and is distinct from mean staining intensity.

### 2.5. Statistical Analysis

To estimate the minimum required sample size, Epi Info version 7.2 was used for case–control studies, along with values reported in previous research for the variables of interest. For immunohistochemical analyses, non-probabilistic convenience sampling was used (*n* = 9). All analyses were performed in Prism version 9.0 (GraphPad Software, San Diego, CA, USA). Continuous variables were summarized as the median and interquartile range (IQR), where IQR is defined as the scalar difference between the 75th and 25th percentiles (IQR = Q3 − Q1). Categorical variables were presented as frequencies and percentages. Before selecting comparison tests, the data were assessed for normality using the Shapiro–Wilk test and Quantile-Quantile plots. Nonparametric statistical tests, including Fisher’s exact test, Chi-Square test, Mann–Whitney test, Wilcoxon matched-pairs signed-rank test, and Kruskal–Wallis test (with Dunn’s post hoc when *p* < 0.05), were used for group comparisons. Correlation analyses among continuous parameters were conducted in the complete SLE cohort (*n* = 40) using two-tailed Spearman rank correlation tests. To control for false discoveries, both unadjusted *p*-values and FDR-adjusted *q*-values (Benjamini–Hochberg procedure) were reported. Associations with *q* < 0.05 were considered statistically robust after multiplicity correction. Associations with nominal *p* < 0.05 but marginal FDR values (0.05 < *q* < 0.10) were interpreted cautiously as preliminary, hypothesis-generating trends. Statistical tests were evaluated at an alpha level of 0.05. Outlier analysis was performed to identify extreme values by the ROUT method, and all values included in the analysis passed this examination.

## 3. Results

### 3.1. Demographic and Clinical Characteristics of Participants

Table 1 describes the demographic and clinical characteristics of participants. The sample comprised participants of similar age and sex: 40 SLE and 30 healthy controls (HC) [age (HC: 30.5, 13; SLE: 41, 20, *p* = 0.06); sex (HC: F = 26, M = 4; SLE: F = 36, M = 4)]. SLE patients were under a regular treatment scheme, including prednisone, antimalarials, and immunosuppressant drugs. The main affected domain was renal (37.5%), followed by hematological (27.5%) and constitutional (25%). The disease activity index, measured by the Systemic Lupus Erythematosus Disease Activity Index 2000 score (SLEDAI-2K), had a mean of 4.25 and a range of 0−32. In patients with renal involvement and available renal biopsies, proliferative and mixed-type classes of LN were identified (Table 1).

### 3.2. Frequency of Expression of CCR2^+^ and CCR5^+^ in Tph Cells

The frequency of Tph cells in SLE patients was higher than in HC (*p* < 0.001), with 31.20, 12.78% (median, IQR) versus 10.93, 6.87% (Figure 1a). The frequency of Tph cells was also increased in SLE patients with clearly active disease and mildly/non-active disease (31.00, 15.45% and 29.60, 14.80%; *p* < 0.05), as well as in SLE patients with or without LN, when compared to HC (Figure 1b,c). A high frequency of CCR5^+^Tph cells was observed in SLE patients; however, CCR2^+^CCR5^+^Tph cells were lower than in HC (Figure 1d). The group of SLE patients with LN (−) had an increased frequency of CCR5^+^Tph cells; however, when frequencies were compared according to LN class, a higher frequency of CCR5^+^Tph cells was observed in those patients who had proliferative LN (classes III and IV) compared to mixed classes (class IV/V) (17.60, 13.50% versus 11.10, 5.24%; *p* < 0.05) (Figure 1e,f).

### 3.3. Frequency of Expression of CCR2^+^ and CCR5^+^ in cTfh Cells

The frequency of cTfh cells in SLE patients was higher than in HC (*p* < 0.001), with 11.20, 4.18% (median, IQR) versus 5.54, 4.23% (Figure 2a). Also, the frequency of cTfh cells was increased in SLE patients with clearly active disease and mildly/nonactive disease (11.00, 3.12% and 9.12, 4.83%; *p* < 0.05) when compared to HC (Figure 2b). As in Tph cells, similar frequencies were observed between LN class comparisons (Figure 2c). Regarding chemokine receptor expression, a higher frequency of CCR2^+^cTfh cells was observed in SLE patients (7.67, 6.03% versus 6.15, 5.72%; *p* < 0.05) (Figure 2d). No differences were observed in the frequencies of CCR2^+^ or CCR5^+^ cTfh cells among SLE patients according to LN groups (Figure 2e); however, CCR5^+^cTfh cells were higher in LN patients with proliferative versus mixed classes (6.56, 9.03% versus 2.23, 2.38%; *p* < 0.05) (Figure 2f).

### 3.4. Serum Levels of IL-6, IL-17, IL-21, TGFβ-3, and CXCL13

Three of the five assessed cytokines were increased in the serum of SLE patients: IL-6, TGFβ-3, and CXCL13 (*p* < 0.05) (Figure 3a). For IL-6, non-significant differences were found between SLE groups (LN (−) and LN (+)) (Figure 3b). However, serum concentrations of TGFβ-3 and CXCL13 were increased in LN (+) versus HC (*p* < 0.05) (Figure 3b). Regarding IL-21 and IL-17, no differences were found in the analyzed groups (Figure 3a,b). Linear correlations were tested between cytokines, and a positive and significant association between CXCL13 and IL-21 serum levels was found (Rho = 0.285, *p* < 0.05) (Figure 3c).

### 3.5. Association of Disease Activity, Cellular Frequency, and Serum Level of IL-21

To evaluate potential associations among activity indices, the chronicity index, and cell frequencies, we performed a matrix correlation analysis and plotted the relevant correlations individually (Figure 4a–c). No significant correlations were found between the disease activity score and cellular frequencies (Figure 4a). The frequency of CCR5^+^Tph decreases as CCR2^+^Tph increases, retaining strong statistical significance after FDR adjustment (Rho = −0.760, *p* < 0.05, *q* < 0.05) (Figure 4b). The frequency of CCR5^+^cTfh shows a non-significant trend toward a decrease with increasing CCR2^+^cTfh (Rho = −0.285, *p* > 0.05, *q* > 0.05) (Figure 4b). However, the frequency of CCR2^+^Tph cells shows a non-significant increasing tendency as the frequency of CCR2^+^cTfh cells increases (Rho = 0.289, *p* > 0.05, *q* > 0.05), and in peripheral blood, Tph cells were more frequent than cTfh cells (Figure 4c). Additionally, we evaluated the potential relationship between cytokines and cell frequencies, and the results showed a linear relationship between serum IL-21 and CCR2^+^CCR5^+^Tph (Rho = 0.344, *p* = 0.03, *q* = 0.06). In contrast, the correlation between CCR2^+^CCR5^+^cTfh and serum IL-21 levels was non-significant (Rho = −0.281, *p* > 0.05, *q* > 0.05) (Figure 4d).

### 3.6. Histological Features of NL

The study analyzed nine LN cases. The median number of glomeruli obtained from percutaneous biopsy was 26, 14.5 (median, IQR). The distribution of cases across LN classes was as follows: Class III and Class IV, 5 cases (55%); and Class IV/V (mixed), 4 cases (45%). Immunoglobulin positivity results were as follows: IgG, 100%; IgA and IgM (both), 89%; C1q, 11.1%; C3c, 100%; and C4c, 22%. Only 11.1% of the cases displayed a full-house pattern. NIH histological scores were 7, 12 (median, IQR) for activity and 4, 3 (median, IQR) for chronicity.

### 3.7. CD4, CXCR5, PD1, CCR5, and CCR2 Expression in LN

We evaluated the tissue expression of CD4, CXCR5, PD1, CCR5, and CCR2 when the biopsy was available. All cases showed positive expression of these markers. Both resident cells (such as mesangial cells, podocytes, Bowman’s Capsule cells, and tubular cells) and inflammatory cells showed expression (Supplementary Figure S2a). Overall analysis showed higher expression of CXCR5 (18.76, 25.10%) than the other markers (CD4: 10.53, 20.03%; PD1: 0.69, 8.53%; CCR5: 8.79, 7.81%; CCR2: 10.1, 16.66%), with no significant differences (*p* > 0.05).

### 3.8. CD4, CXCR5, PD1, CCR5, and CCR2 Expression by LN Class

Subsequently, we compared marker expression across LN classes. Regarding the expression of CD4 (III: 9.71, 16.21% vs. IV: 14.02, 27.86% vs. mixed: 20.71, 17.2%; *p* > 0.05), PD1 (III: 0.69, 3.27% vs. IV: 11.5, 13.88% vs. mixed: 0.32, 0.87%; *p* > 0.05), CXCR5 (III: 17.94, 26.15% vs. IV: 25.29, 39.2% vs mixed: 12.39, 25.23%; *p* = 0.105), CCR5 (III: 8.68, 11.89% vs. IV: 7.93, 3.68% vs. mixed: 9.03, 9.17%; *p* = 0.259), and CCR2 (III: 9.54, 11.31% vs. IV: 18.35, 19.14% vs. mixed: 8.06, 19.51%; *p* = 0.088), they did not show statistical differences.

### 3.9. Expression of CD4, CXCR5, PD 1, CCR5, and CCR2 According to Histological Zones

We also compared marker expression between the glomerular (Glom) and tubulo-interstitial (TubInt) zones. CD4 showed higher expression in the tubulo-interstitial zone than in the glomerular zone (Glom: 0.00, 5.88% vs. TubInt: 17.42, 19.35%; *p* = 0.0011) (Supplementary Figure S2b). Regarding CCR5 (Glom: 6.99, 4.85% vs. TubInt: 10.28, 14.14%; *p* > 0.05) and CCR2 (Glom: 8.26, 11.24% vs. TubInt: 10.72, 19.03%; *p* > 0.05), they did not show statistical differences according to histological zones (Supplementary Figure S2c,d).

### 3.10. Association Between NIH Histological Predominance and CD4, CXCR5, PD1, CCR5, and CCR2 Expression

Finally, we categorized LN cases by NIH histological score based on whether activity (Act) or chronicity (Chro) predominated, then compared marker expression. CD4 showed higher expression in tissues with predominantly chronic lesions (Act: 7.36, 16.20% vs. Chro: 24.77, 7.60%; *p* = 0.0476) (Supplementary Figure S2e). Regarding CCR5 (Act: 7.83, 3.63% vs. Chro: 9.20, 16.75%; *p* > 0.05) and CCR2 (Act: 9.30, 7.21% vs. Chro: 15.20, 39.36%; *p* > 0.05), they did not show statistical differences according to the NIH histological score (Supplementary Figure S2f,g).

## 4. Discussion

The immunopathology of SLE involves T helper subpopulations that collaborate with B cells, facilitating their differentiation into antibody-producing cells [16]. Recently, the expansion of a unique inflammatory T helper subpopulation, Tph cells, has been described in SLE and other autoimmune diseases [8,9,17]. The absence of CXCR5 on Tph cells suggests their potential to migrate to inflamed tissues and collaborate with B cells at sites of organ damage [18]. In our study, we found a higher frequency of Tph and cTfh cells in SLE compared with HC, but no significant differences were observed when stratified by disease activity or between LN (−) and LN (+) groups.

The role of Tph cells in the formation of TLS, associated with increased disease activity, has been reported previously, highlighting the involvement of migration molecules such as the chemokine receptors CCR2 and CCR5 [9,10]. To investigate the presence and potential association of these chemokine receptors with Tph and cTfh cells, we determined their frequencies by flow cytometry. We found increases in both molecules when comparing HC and LN(−)/LN(+) SLE patients.

The role of CCR2 in T helper cells was studied in the early 2000s, indicating a relationship between reductions in CCR2^+^CD4^+^ T cells and SLE flares [19]. In addition, CCR2 has been specifically implicated in kidney damage, as CCR2 deficiency (in Ccr2-deficient MRL/lpr mice) reduces T-cell infiltration into the kidney [20]. CCR2 expression on multiple T cell populations infiltrating the rheumatoid arthritis synovium, including Tph cells, has also been reported [21]. In patients with chronic Graft-Versus-Host Disease (cGVHD), Tph cells and CCR2^+^Tph cells are increased compared with those in patients without cGVHD [22]. Here, we identify a significant reduction in CCR2^+^CCR5^+^Tph cells in SLE patients compared with HC; however, no significant differences were observed between the LN (+) and LN (−) groups.

Meanwhile, in CCR2^+^cTfh cells, we observed an increase in SLE compared with HC. These findings support the idea of specific CCR2 expression across different T helper subpopulations in SLE. Previous evidence suggests that, during inflammation, high levels of CCR2 and CCL2 are expressed in inflamed tissue, guiding T cells toward inflammatory phenotypes [23,24]. A key finding of this study is the unexpected reduction in the frequency of the CCR2^+^CCR5^+^Tph subset in SLE patients compared with HC. In SLE, the consistent upregulation of CCL2 and CCL5, the primary ligands for these receptors in the kidneys, vascular endothelium, and peripheral blood, suggests that these cells are preferentially mobilized from the circulation toward sites of active inflammation [10,24,25,26].

The histopathological data from our renal biopsies provide further insight into this phenomenon. While CCR5^+^Tph frequencies were comparable between SLE patients with and without LN, a distinct enrichment was observed in pure proliferative classes (III and IV) compared to mixed classes (IV/V). Our results showed an increased frequency of CCR2^+^Tph, CCR5^+^Tph, and CCR2^+^CCR5^+^Tph cells compared with their counterparts in cTfh cells from SLE patients, consistent with previous reports [27,28]. These findings support that migration to inflamed tissues is guided by these chemokines, which has been proposed previously ([9,29]) by the demonstration of CCR2^+^CD4^+^T cells and CCR5^+^CD4^+^T cells and their ligands (CCL2/CCR2 and CCL5/CCR5) in the labial gland tissue of patients with primary Sjogren Syndrome, suggesting that Tph cells may be recruited to the salivary glands through the CCR2/CCL2 or CCR5/CCL5 axes, and then participate in salivary gland lymphocyte infiltration and inflammation. Interestingly, CCR5 blockade has been proposed as a potential therapy for LN [10], and the presence of Tph cells in human lupus nephritis samples was previously confirmed by single-cell RNA sequencing, which identified a cluster with higher expression of PRDM1 and CCL5 [30,31]. Whereas CCR5 expression is elevated on circulating pathogenic T cells in SLE, experimental models demonstrate that CCR5 deficiency can paradoxically aggravate lupus nephritis, highlighting the complex cell-type-specific roles of chemokine receptors in tissue recruitment versus local regulation [32].

On the other hand, many cytokines have been investigated in the serum of patients with SLE, particularly during SLE flares [33,34]. We found significant increases in serum levels of IL-6, TGF-β3, and CXCL13, accompanied by an elevated frequency of cTfh and Tph cells in SLE patients compared with HC. The increase in serum IL-6 levels has been reported previously in SLE and other autoimmune conditions, and its role as an inducer cytokine of the Tfh phenotype is well documented [35,36]. Recently, the description of the combination of IL-6 with TGF-β for the induction of the Tfh phenotype in vitro, and more importantly, the involvement of an isoform, TGF-β3, has been implicated in the acquisition of the Tph phenotype [37,38], supporting the inducer role of IL-6 and TGF-β3 in the cTfh and Tph populations. We also measured serum levels of IL-17 and IL-21 because their roles as effector cytokines in cTfh and, especially, Tph cells in lupus and LN have been reported previously [7,39]. Still, no significant differences were observed in our sample. Previous longitudinal studies have established that immunosuppressive therapy and glucocorticoids generally suppress circulating Tph/cTfh cell frequencies and reduce systemic CXCL13/IL-21 levels in SLE [40]. Therefore, the expansion of Tph cells and elevated cytokine levels detected in our cohort occurred despite ongoing pharmacological treatment, reinforcing the biological relevance of these immunological alterations in active SLE.

Furthermore, serum levels of CXCL13, another important Tph effector cytokine, were increased in SLE and correlated with IL-21. The role of CXCL13 in SLE as an effector cytokine of Tph has been reported by our group and others, highlighting its presence in SLE accompanied by lupus nephritis [39,41,42,43]. Previously, inconsistent results regarding IL-21 levels and their association with disease activity have been reported, which may be attributed to heterogeneity in the clinical features of patients with SLE, including disease evolution and disease activity levels, as well as different stratifications of disease activity indexes [44]. Although we did not find a significant increase in IL-21 serum levels, its positive correlation with CXCL13 serum levels is consistent with a conservative role for IL-21, with a more renal domain-related increase in CXCL13. IL-21 is the signature cytokine of the Tfh/Tph subpopulations, as it facilitates the differentiation of B cells into autoantibody-producing cells. This evidence could imply that the simultaneous increase in both cytokines is related to the recruitment of effector cells to the inflamed tissue, with CXCL13 being more important in patients with LN. In this regard, CXCL13 has been proposed as a biomarker for SLE and LN [42], and the relationship between CXCL13 levels in LN and tertiary lymphoid structures has been proposed [45,46,47].

Additionally, the mediating role of CCR2 in the expression of inflammatory T-cell cytokines and its role in modulating the effector/regulatory T-cell ratio have been proposed previously [18]. In our sample, we did not find any significant correlation between serum cytokine levels and CCR2^+^cTfh or CCR2^+^Tph, but we observed a significant correlation with IL-21 in double-positive CCR2^+^CCR5^+^Tph cells (Rho = 0.344, *p* = 0.03, *q* = 0.06). Although these trends must be interpreted with caution due to multiple testing considerations, they warrant further validation in prospective cohorts. The complexity of Tph cell trafficking is further highlighted by the robust negative correlation observed between CCR5^+^Tph and CCR2^+^Tph subsets (Rho = −0.760, *p* < 0.01, *q* < 0.01). This reciprocal relationship suggests a divergent functional commitment within the circulating Tph pool. One possibility is a hierarchical affinity for tissue recruitment, in which the CCR2 axis might prioritize rapid extravasation. At the same time, the expansion of the CCR5^+^ subset reflects a distinct proliferative state associated with sustained local inflammation. Alternatively, this inverse correlation could indicate a phenotypic switch during Tph maturation, in which the loss of CCR2 and the acquisition of CCR5 mark a transition toward a more specialized “tissue-resident” effector profile, specifically tailored to the microenvironment of the proliferative lupus kidney. Additionally, because these fractions are mutually exclusive and expressed as percentages of a shared parent T cell population, the observed negative association may partly reflect the inherent compositional structure of relative proportions rather than definitive evidence of biological phenotypic switching or differential tissue recruitment. Future studies employing single-cell functional tracking will be necessary to determine whether these subsets undergo active transdifferentiation or exhibit distinct tissue homing dynamics.

Based on these observations, we propose two potential, non-exclusive pathways:

- The Pathological Continuum Hypothesis: The expansion of CCR5^+^Tph cells may serve as an early, non-specific marker of disease flares and active proliferative LN. In this model, the initial proliferative phase of the disease triggers a robust expansion of CCR5^+^Tph cells, which are then progressively recruited to the renal parenchyma.
- Cytokine-Driven Phenotypic Polarization Hypothesis: The specific microenvironment, characterized by elevated serum levels of IL-6, TGFβ-3, and CXCL13, may selectively favor the differentiation or survival of the CCR5^+^Tph lineage over other subsets. This cytokine interplay likely modulates the homing capacity of Tph cells, aligning their migratory profile with the chemokine gradients present in the lupus-affected kidney.

Altogether, the findings presented in this work confirm the expression of CCR2 and CCR5 in cTfh and Tph cells from SLE patients and suggest their involvement in LN, showing a correlation between serum IL-21 levels and CCR2 and CCR5 expression in Tph cells. The observational associations presented support the possibility of an inflammatory cytokine/cell axis that may redirect Tph cells in patients with renal involvement in SLE. We acknowledge that these findings should be interpreted with caution, as cytokine levels were measured in serum and may not reflect local cytokine concentrations within renal tissue, and the statistically significant cytokine–cell correlations were of moderate strength. Beyond their mechanistic implications, these distinct chemokine receptor expression patterns may serve as exploratory immunophenotypic biomarkers in LN. Based on the observed associations, co-expression of CCR2 and CCR5 on pathogenic helper T cell subsets could offer non-invasive insights into renal immune activity.

Our work has methodological limitations: we did not measure B cell populations in PBMCs, nor did we evaluate the presence of cTfh and Tph, or cytokines, in situ by histology of renal biopsies to perform orthogonal analysis and correlate circulating cellular frequencies with renal tissue colocalization studies for our markers of interest (CD4, PD1, CXCR5, CCR2, and CCR5). Additionally, we did not conduct participant follow-up, and our correlations cannot establish causality. The sampling was consecutive and non-paired, so we may not find significant differences in some cases, for example, in serum levels of IL-21, in part due to the sample size and the lack of heterogeneity in clinical domains beyond renal or lupus nephritis. Several limitations regarding the tissue-level analyses must be emphasized. First, given the relatively small sample size of renal biopsies and the absence of statistically significant differences between LN (+) and LN (−) subgroups across secondary parameters, our tissue findings should be interpreted strictly within a descriptive and exploratory framework. Second, because our cross-sectional study design captures only a single timepoint, these observations cannot establish active cellular trafficking dynamics or predict clinical disease flares. Consequently, these expression profiles represent preliminary candidate patterns that require prospective validation.

## 5. Conclusions

Although the absence of in situ tissue co-localization is a limitation of this study, our findings provide valuable insights into the role of Tph cells in LN. Future studies incorporating B-cell profiling, renal biopsy histology with immunofluorescence, and longitudinal patient follow-up will be essential to elucidate the potential cytokine–Tph–CCR axis and its contribution to LN pathogenesis.

## Figures and Tables

**Figure 1:**
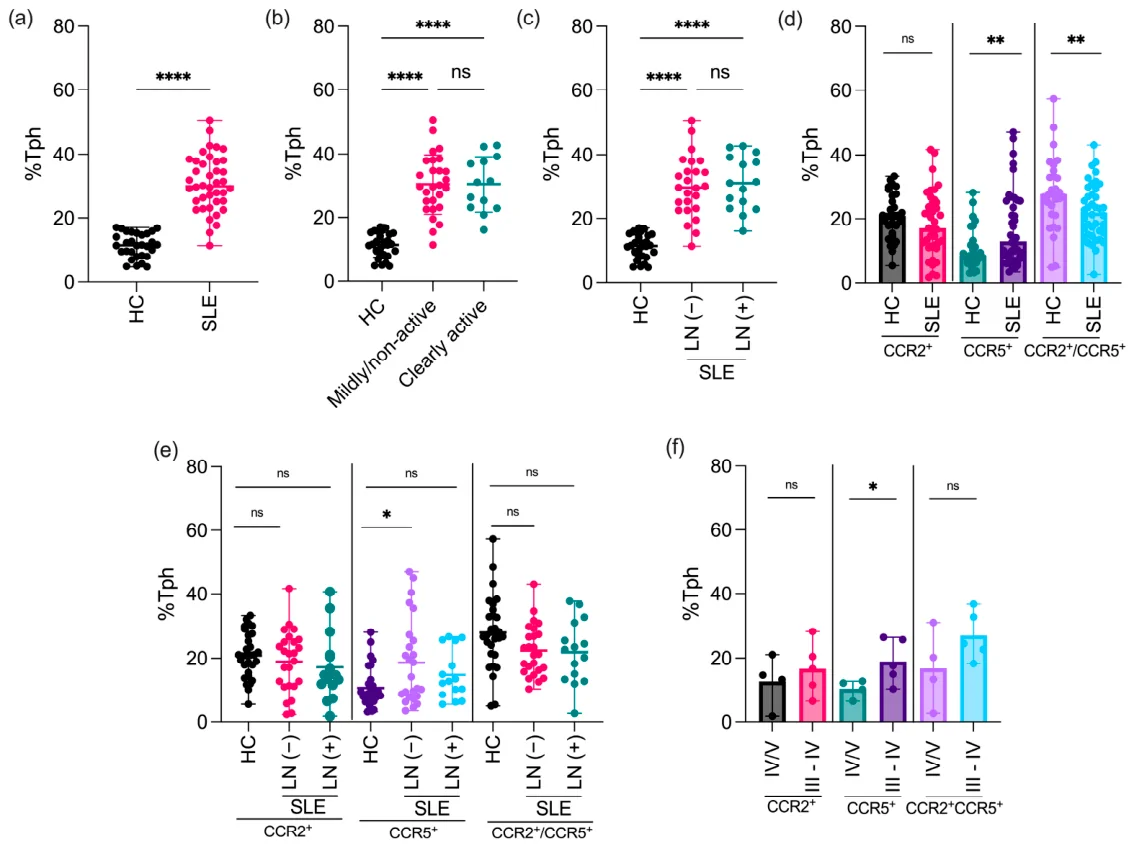
Frequency of Tph cells in patients with SLE. (**a**) Frequency of Tph cells in HC (*n* = 30) and SLE (*n* = 40), Mann–Whitney test. (**b**) Frequency of Tph cells in HC (*n* = 30), Mildly/non-active SLE (*n* = 27), and clearly active SLE (*n* = 13), Kruskal–Wallis test. (**c**) Frequency of Tph cells in HC (*n* = 30), LN (−) SLE (*n* = 25), and LN (+) SLE (*n* = 15), Kruskal–Wallis test. (**d**) Frequency of Tph CCR2^+^, Tph CCR5^+^ and Tph CCR2^+^/CCR5^+^ cells in HC (*n* = 30) and SLE (*n* = 40), Mann–Whitney test. (**e**) Frequency of Tph CCR2^+^, Tph CCR5^+^ and Tph CCR2^+^/CCR5^+^ cells in HC (*n* = 30), LN (−) SLE (*n* = 25), and LN (+) SLE (*n* = 15), Kruskal–Wallis test. (**f**) Frequency of Tph CCR2^+,^ Tph CCR5^+^, and Tph CCR2^+^/CCR5^+^ cells in IV/V (*n* = 5), III-IV (*n* = 4) LN classes, Kruskal–Wallis test. Statistically significant differences: non-significant: ns, *p* < 0.05 *, *p* < 0.01 **, *p* < 0.0001 ****. Tph: T peripheral helper cells; CCR2: C-C chemokine receptor 2; CCR5: C-C chemokine receptor 5; HC: Healthy Controls; SLE: Systemic Lupus Erythematosus patients; Non/Mildly active: SLEDAI-2K ≤ 5; Clearly active: SLEDAI-2K > 5; LN (+): Lupus nephritis SLE; LN (−): Without lupus nephritis SLE patients.

**Figure 2:**
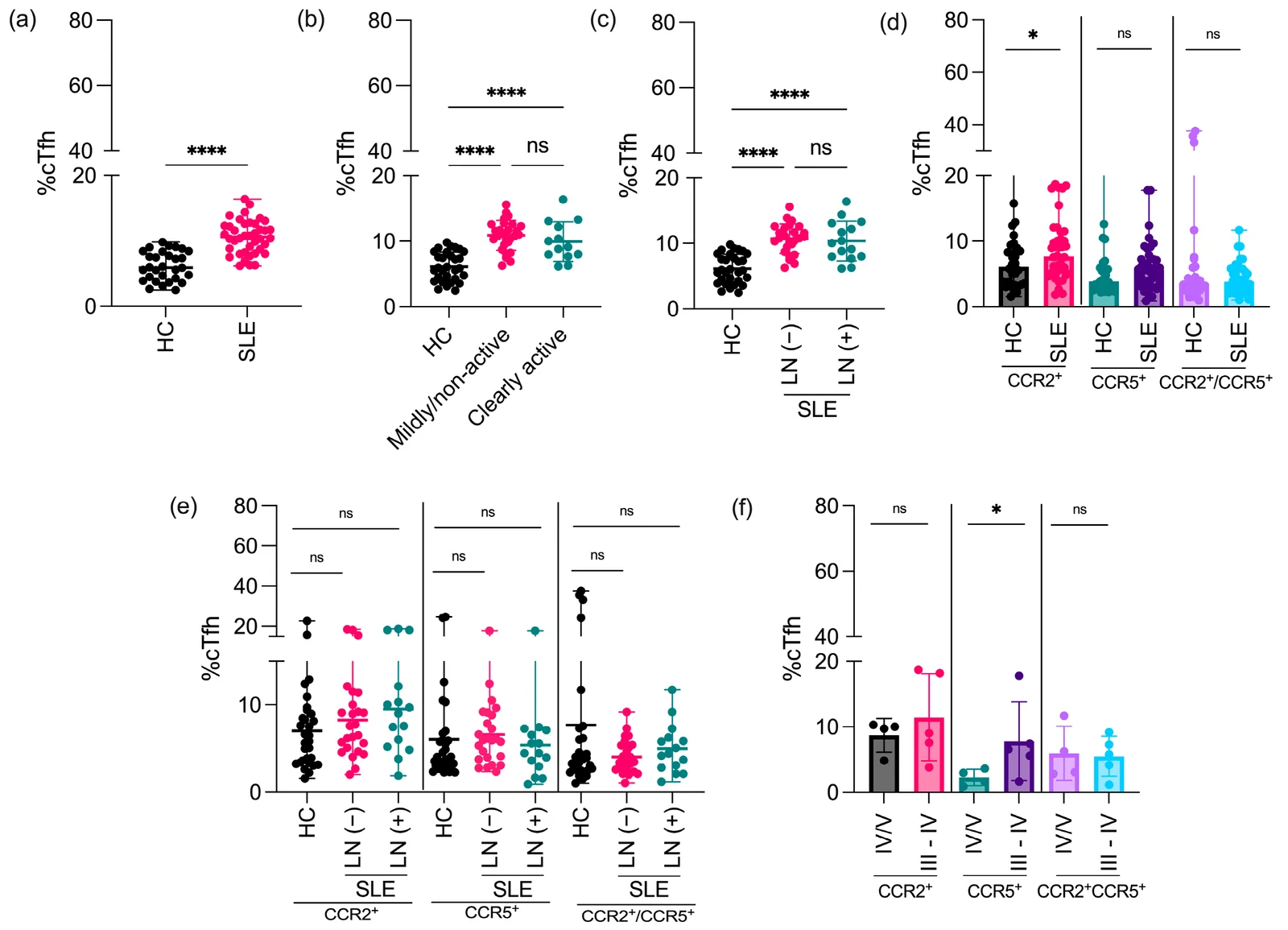
Frequency of cTfh cells in patients with SLE. (**a**) Frequency of cTfh cells in HC (*n* = 30) and SLE (*n* = 40), Mann–Whitney test. (**b**) Frequency of cTfh cells in HC (*n* = 30), Mildly/non-active SLE (*n* = 27), and clearly active SLE (*n* = 13), Kruskal–Wallis test. (**c**) Frequency of cTfh cells in HC (*n* = 30), LN (−) SLE (*n* = 25), and LN (+) SLE (*n* = 15), Kruskal–Wallis test. (**d**) Frequency of cTfh CCR2^+^, cTfh CCR5^+^ and cTfh CCR2^+^/CCR5^+^ cells in HC (*n* = 30) and SLE (*n* = 40), Mann–Whitney test. (**e**) Frequency of Tph CCR2^+^, Tph CCR5^+^ and Tph CCR2^+^/CCR5^+^ cells in HC (*n* = 30), LN (−) SLE (*n* = 25), and LN (+) SLE (*n* = 15), Kruskal–Wallis test. (**f**) Frequency of cTfh CCR2^+,^ cTfh CCR5^+^, and cTfh CCR2^+^/CCR5^+^ cells in IV/V (*n* = 5), III-IV (*n*= 4) LN classes, Kruskal–Wallis test. Statistically significant differences: non-significant: ns, *p* < 0.05 *, *p* < 0.0001 ****. cTfh: circulant T follicular helper cells; CCR2: C-C chemokine receptor 2; CCR5: C-C chemokine receptor 5; HC: Healthy Controls; SLE: Systemic Lupus Erythematosus patients; Non/Mildly active: SLEDAI-2K ≤ 5; Clearly active: SLEDAI-2K > 5; LN (+): Lupus nephritis SLE; LN (−): Without lupus nephritis SLE patients.

**Figure 3:**
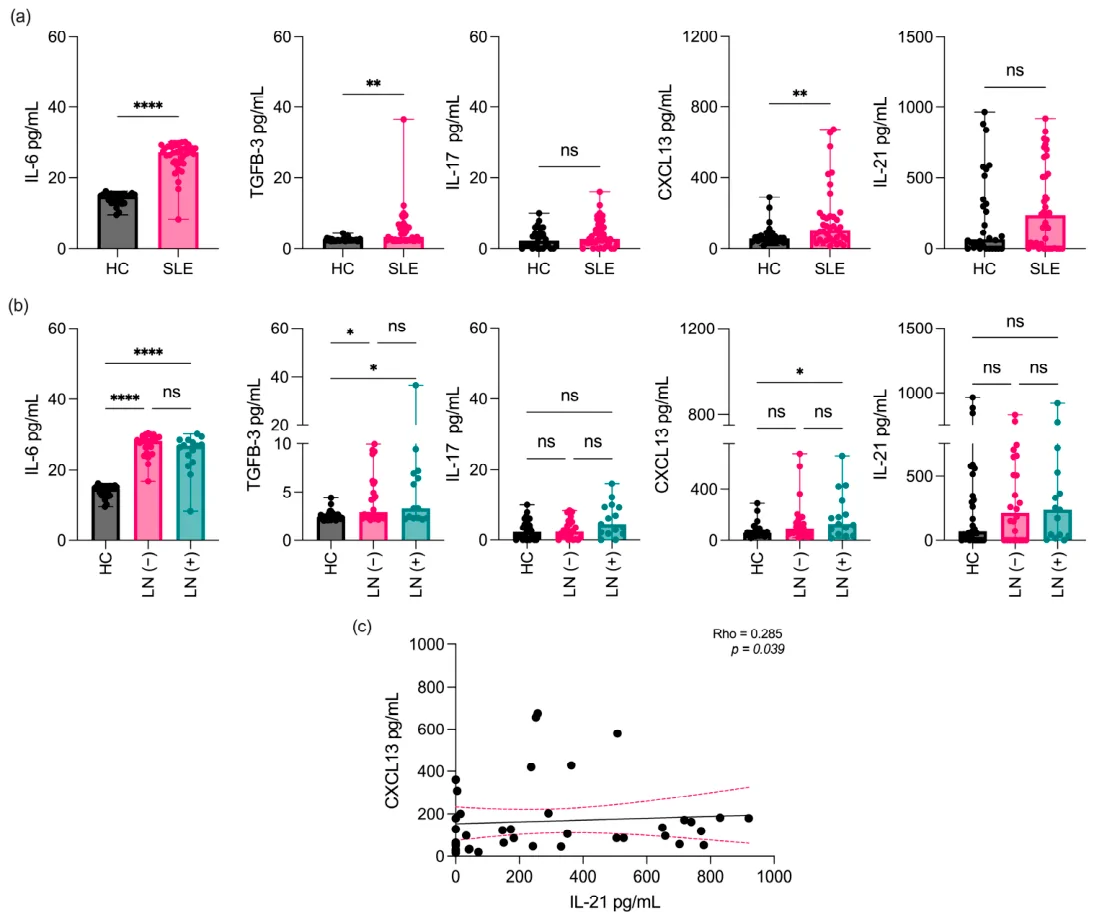
Serum concentrations of inducer and effector cytokines in patients with SLE. (**a**) Serum concentrations of IL-6, TGFβ-3, IL-17, CXCL13, and IL-21 (pg/mL) in HC (n = 30), SLE (n = 40), Mann–Whitney’s test. (**b**) Serum concentrations of IL-6, TGFβ-3, IL-17, CXCL13, and IL-21 (pg/mL) in HC (*n* = 30), LN (−) SLE (*n* = 25), and LN (+) SLE (*n* = 15), Kruskal–Wallis test. (**c**) Correlation between serum concentrations of IL-21 and CXCL13, Spearman test. Statistically significant differences: non-significant: ns, *p* < 0.05 *, *p* < 0.01 **, *p* < 0.0001 ****. IL-6: Interleukin 6; TGFβ-3: Transforming growth factor beta 3; CXCL13: Chemokine (C-X-C motif) ligand 13; IL-17: Interleukin 17; IL-21: Interleukin 21; HC: Healthy Controls; SLE: Systemic Lupus Erythematosus patients; LN (+): Lupus nephritis SLE; LN (−): Without lupus nephritis SLE patients.

**Figure 4:**
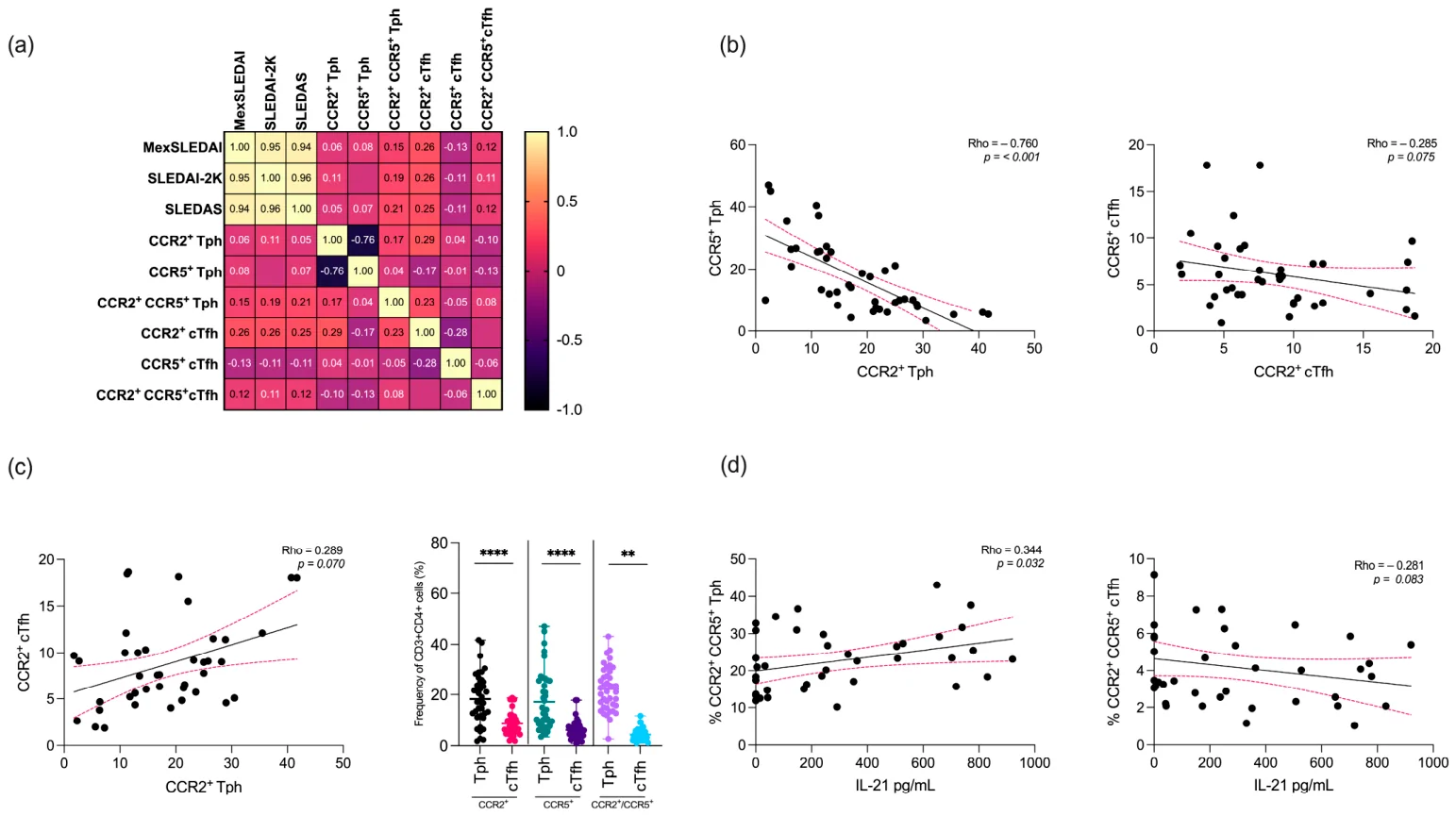
Correlation between cellular frequencies, serum IL-21, and disease activity. (**a**) Heatmap of correlations between cellular frequencies and Mex-SLEDAI, SLEDAI-2K, and SLEDAS, Spearman test (*n* = 40). (**b**) Left: correlation between CCR2^+^Tph cells and CCR5^+^Tph cells; right: CCR2^+^cTfh cells and CCR5^+^cTfh cells, Spearman test (*n* = 40). (**c**) Left: correlation between CCR2^+^cTfh cells and CCR2^+^Tph cells, Spearman test (*n* = 40); right: frequencies of CCR2^+^, CCR5^+^ and CCR2^+^/CCR5^+^Tph and cTfh cells, Wilcoxon matched-pairs signed-rank test (*n* = 40). (**d**) left: correlation between IL-21 and CCR2^+^/CCR5^+^Tph cells; right: CCR2^+^/CCR5^+^cTfh cells, Spearman test (*n* = 40). Statistically significant differences: non, *p* < 0.01 **, *p* < 0.0001 ****. Tph: T peripheral helper cells; cTfh: circulant T follicular helper cells; CCR2: C-C chemokine receptor 2; CCR5: C-C chemokine receptor 5; IL-21: Interleukin 21; HC: Healthy Controls; SLE: Systemic Lupus Erythematosus patients; SLEDAI-2K: Systemic Lupus Erythematosus Disease Activity Index 2000; Mex-SLEDAI: Mexican Modification of the Systemic Lupus Erythematosus Disease Activity Index; SLEDAS: Systemic Lupus Erythematosus Disease Activity Score.

**Table 1:** Demographic and clinical characteristics.

Variable	SLE (n = 40)	HC (n = 30)
Age, years	41.0, 20	30.5, 13
Female/Male (*n*)	36.0/4.0	26.0/4.0
BMI, kg/m^2^	26.4, 6.0	27.1, 6.8
Clinical characteristics		
Disease evolution, years	10.0, 7.2	-
Mex-SLEDAI	1.0, 7.2	-
SLE-DAS	0.37, 7.9	-
SLEDAI-2K	0.50, 6.0	-
Non/Mildly active ^1^; % (*n*)	67.5% (27)	-
Clearly active ^1^; % (*n*)	32.5% (13)	-
ANA (+) >1:80; % (*n*)	100.0% (40)	-
Clinical domains ^2^		
Constitutional; % (*n*)	25.0% (10)	-
Renal; % (*n*)	37.5% (15)	-
Mucocutaneous; % (*n*)	22.5% (9)	-
Musculoskeletal; % (*n*)	15.0% (6)	-
Hematological; % (*n*)	27.5% (11)	-
Pharmacological therapy ^3^		
Prednisone; % (*n*)	52.6% (21)	-
Antimalarials; % (*n*)	67.5% (27)	-
Immunosuppressant; % (*n*)	77.5% (31)	-
Lupus nephritis class ^4^		
Proliferative classes (IV and III), % (*n*)	55% (5)	-
Mixed Type (IV/V), % (*n*)	45% (4)	-

HC: Healthy Controls; SLE: Systemic Lupus Erythematosus patients; BMI: Body Mass Index; SLEDAI-2K: Systemic Lupus Erythematosus Disease Activity Index 2000; Mex-SLEDAI: Mexican Modification of the Systemic Lupus Erythematosus Disease Activity Index; SLEDAS: Systemic Lupus Erythematosus Disease Activity Score. Quantitative data are shown as median, IQR. ^1^ Non/Mildly active (SLEDAI-2K ≤ 5); Clearly active (SLEDAI-2K > 5). ^2^ Constitutional: fatigue; Renal: persistent proteinuria > 0.5 g/24 h; Mucocutaneous: non-scarring alopecia, oral ulcers; Musculoskeletal: joint involvement; Hematological: leukopenia. ^3^ Antimalarial drugs include chloroquine or hydroxychloroquine. Immunosuppressive treatment includes azathioprine, mycophenolate mofetil, methotrexate, or cyclophosphamide. ^4^ Immunofluorescence was employed for LN classification of biopsies of renal tissue.

## Data Availability

The original contributions presented in this study are included in the article/Supplementary Materials. Further inquiries can be directed to the corresponding author.

## Supplementary Materials

The following supporting information can be downloaded at https://www.mdpi.com/article/10.3390/diagnostics16213687/s1: Table S1: Flow Cytometry Antibodies; Figure S1: Flow Cytometry Strategy; Figure S2: Representative cases of expression of CD4, CXCR5, PD1, CCR5, and CCR2 in LN cases.

## Abbreviations

The following abbreviations are used in this manuscript:

cTfh	Circulating T follicular helper cells
Tph	T peripheral helper cells
SLE	Systemic Lupus Erythematosus
CCR2	Chemokine receptor 2
CCR5	Chemokine receptor 5
LN	Lupus nephritis
HC	Healthy controls
ELISA	Enzyme-Linked Immunosorbent Assay
SLEDAI-2K	Systemic Lupus Erythematosus Disease Activity Index 2000
Mex-SLEDAI	Mexican Systemic Lupus Erythematosus Disease Activity Index
CD4	Cluster of Differentiation 4
CXCR5	C-X-C motif chemokine receptor 5
PD1	Programmed Cell Death Protein 1
IL-6	Interleukin-6
IL-17	Interleukin-17
IL-21	Interleukin-21
TGF-β3	Transforming Growth Factor-beta 3
CXCL13	C-X-C motif chemokine ligand 13
TLS	Tertiary lymphoid structures
EDTA	Ethylenediaminetetraacetic acid
PBMC	Peripheral Blood Mononuclear Cells
cGVHD	Chronic Graft-Versus-Host Disease
CCL2	Chemokine (C-C motif) Ligand 2
CCL5	Chemokine (C-C motif) Ligand 5
