# Supplementary material for: CCR2 and CCR5 Expression on Tph Cells Is Associated with IL-21 and Renal Involvement in Systemic Lupus Erythematosus
Source: Diagnostics (Basel). 2026 Nov 8;16(21):3687. doi: 10.3390/diagnostics16213687 (PMC13763565; doi:10.3390/diagnostics16213687)
Supplement: Supplementary file 1 [file diagnostics-16-03687-s001.zip › diagnostics-4636420-supplementary.pdf]

Supplementary Materials

Supplementary Table S1. Flow Cytometry Antibodies

Fluorochrome - Target	Dilution factor	Catalog number and brand
PERCP - CD3	1:8	317338/ Biolegend
BV510 - CD4	1:4	317408/ Biolegend
AF647- CXCR5	1:64	588113/ BD Pharmingen
PECy7 – PD1	1:4	367414/ Biolegend
PE – CCR2	1:4	357206/Biolegend
APCFire750 – CCR5	1:8	359134/ Biolegend

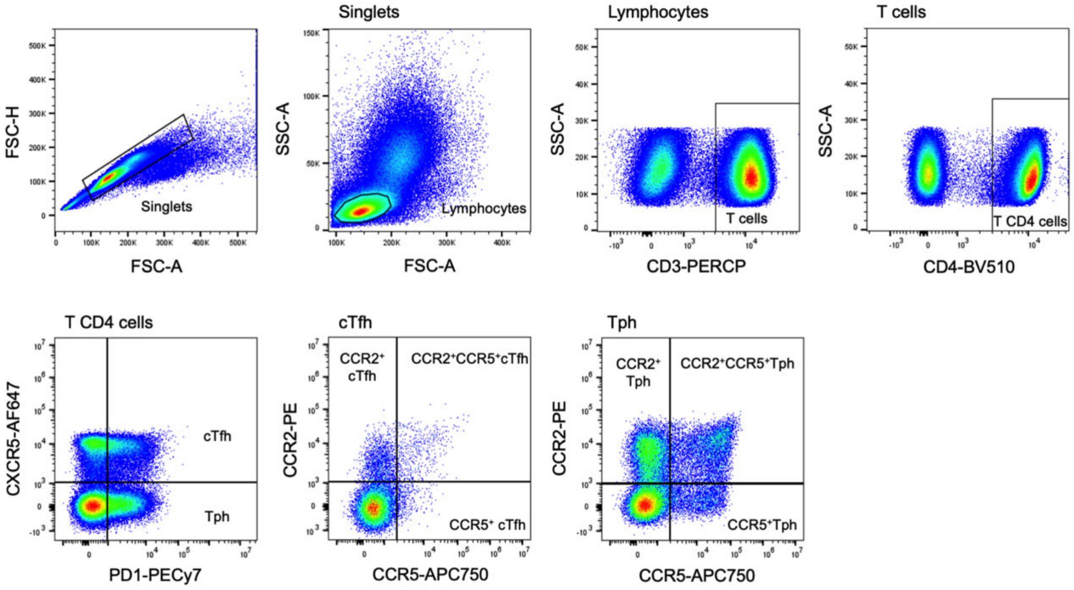

Supplementary Figure S1. Flow Cytometry Strategy. Sequential gating strategy used to identify circulating Tph and cTfh cells and their chemokine receptor profiles. Singlets and lymphocytes were gated based on FSC/SSC, followed by sequential selection of CD3+ T cells and CD4+ T helper cells. Parent Tph (CD4$^+$/CXCR5$^-$/PD-1$^+$) and cTfh (CD4$^+$/CXCR5$^+$/PD-1$^+$) populations were delineated based on CXCR5 and PD-1 expression. Quadrant analysis of CCR2 versus CCR5 co-expression within gated cTfh and Tph cells defined single-positive (CCR2$^+$ or CCR5$^+$) and double-positive (CCR2$^+$CCR5$^+$) subpopulations.

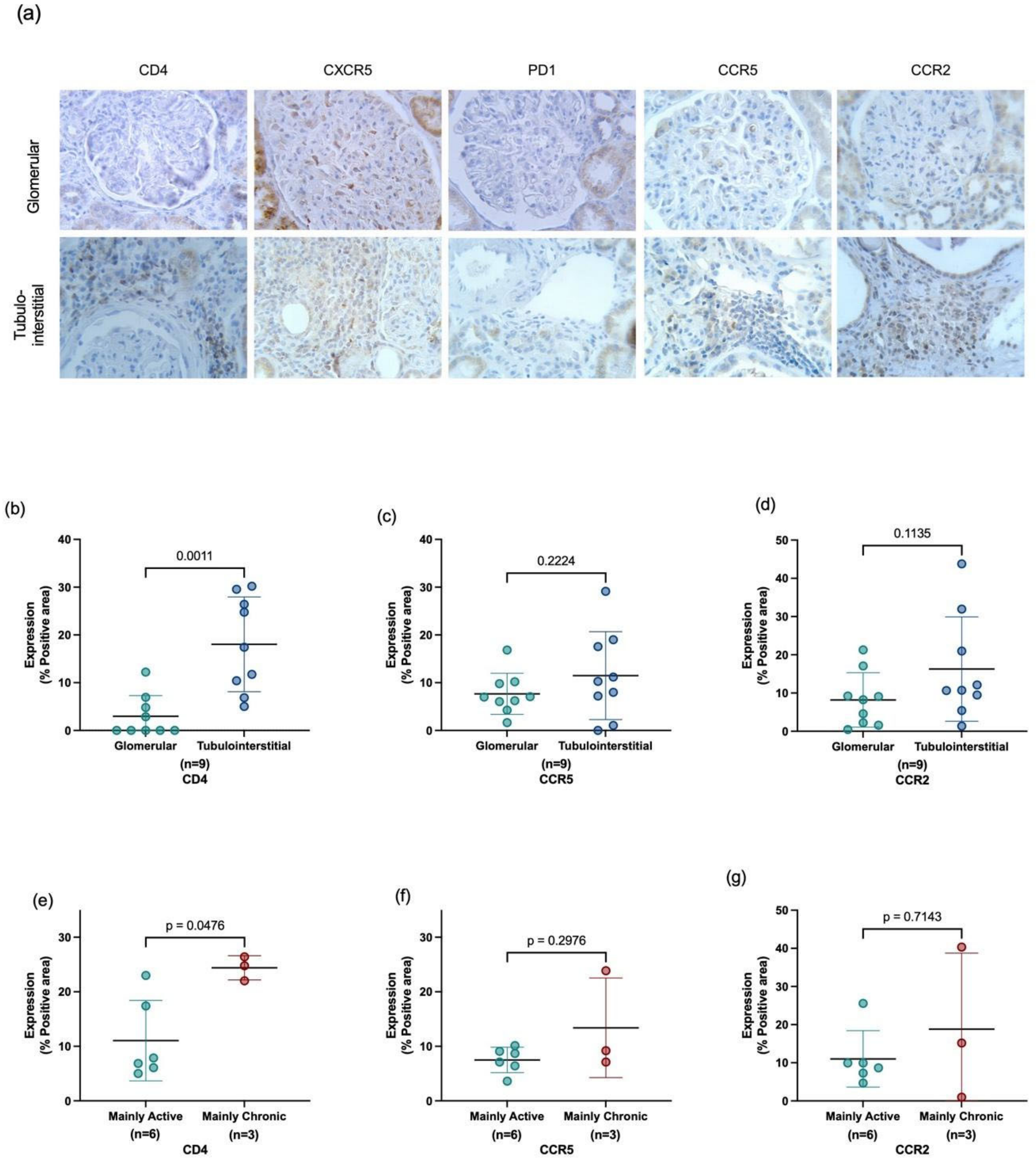

Supplementary Figure S2. Expression of CD4, CXCR5, PD1, CCR5, and CCR2 in kidney tissues of LN cases. Representative cases of expression of CD4, CXCR5, PD1, CCR5, and CCR2 in LN cases. Biomarkers were expressed in glomerular and tubulo-interstitial zones. Both kidney-resident cells (as mesangial cells, podocytes, Bowman's Capsule cells, and tubular cells) and inflammatory cells also expressed them (2a). Comparison of CD4 (n=9), CCR5 (n=9), and CCR2 (n=9) expression according to tissular zones (2b, 2c, and 2d, respectively). Evaluation of CD4, CCR5, and CCR2 (2e, 2f, and 2g, respectively) by predominance of active (n=6) or chronic lesion (n=3) according to NIH score. Data are shown as median and IQR. p-values were obtained using the Mann-Whitney U test or Wilcoxon matched-pairs signed-rank test. LN: lupus nephritis.
